# Rice Photosynthetic Productivity and PSII Photochemistry under Nonflooded Irrigation

**DOI:** 10.1155/2014/839658

**Published:** 2014-03-05

**Authors:** Haibing He, Ru Yang, Biao Jia, Lin Chen, Hua Fan, Jing Cui, Dong Yang, Menglong Li, Fu-Yu Ma

**Affiliations:** ^1^Agricultural College, Shihezi University/Key Laboratory of Oasis Ecology Agriculture of Xinjiang Bingtuan, Shihezi, Xinjiang 832003, China; ^2^Agricultural Drought Research Institute of Tianye Group Company, XinJiang 832003, China

## Abstract

Nonflooded irrigation is an important water-saving rice cultivation technology, but little is known on its photosynthetic mechanism. The aims of this work were to investigate photosynthetic characteristics of rice during grain filling stage under three nonflooded irrigation treatments: furrow irrigation with plastic mulching (FIM), furrow irrigation with nonmulching (FIN), and drip irrigation with plastic mulching (DI). Compared with the conventional flooding (CF) treatment, those grown in the nonflooded irrigation treatments showed lower net photosynthetic rate (*P*_*N*_), lower maximum quantum yield (*F*_*v*_/*F*_*m*_), and lower effective quantum yield of PSII photochemistry (Φ_PSII_). And the poor photosynthetic characteristics in the nonflooded irrigation treatments were mainly attributed to the low total nitrogen content (TNC). Under non-flooded irrigation, the *P*_*N*_, *F*_*v*_/*F*_*m*_, and Φ_PSII_ significantly decreased with a reduction in the soil water potential, but these parameters were rapidly recovered in the DI and FIM treatments when supplementary irrigation was applied. Moreover, The DI treatment always had higher photosynthetic productivity than the FIM and FIN treatments. Grain yield, matter translocation, and dry matter post-anthesis (DMPA) were the highest in the CF treatment, followed by the DI, FIM, and FIN treatments in turn. In conclusion, increasing nitrogen content in leaf of rice plants could be a key factor to improve photosynthetic capacity in nonflooded irrigation.

## 1. Introduction

To meet the increasing demand for rice from the word's growing population, rice production must increase by 60% by 2025 according to current projections [1]. However, a shortage of water resources is already a problem for agriculture [2]. Therefore, water shortages are expected to limit rice production, which requires a large amount of water. In recent years, various water-saving cultivations for rice production have been tested. Rice is grown under nonflooded irrigation with adequate inputs and supplementary irrigation at times during the growth period when rainfall is insufficient. Rice grown in this way shows high water use efficiency and grain yield [3–7]. These technologies provide important theoretical reference values for coping with potential water deficit and the demands for a staple food.

Grain yield formation mainly depends on photosynthetic production during the grain-filling stage [8, 9]. Therefore, photosynthesis during the grain-filling stage is an important physiological factor affecting biomass and grain yield [10]. The flag leaves, the second and third leaves from the top of the plant are considered to be functional leaves during grain filling [11]. Therefore, studies on the photosynthetic characteristics of flag leaves during the grain-filling stage help us understand their physiological status and the grain production potential of the plant [11]. Water stress is the most important limiting factor affecting plant growth and crop production worldwide. Previous studies reported that the photosynthetic rate of the flag leaves declined quickly when grain-filling stage was subjected to nonflooded conditions [12, 13] and that grain yield was affected by water deficit stress [14, 15]. However, studies on photosynthetic mechanisms of rice in nonflooded condition have generally applied only short-term water regulation of water supply, for example, growing rice under nonflooded cultivation during the grain-filling stage, while applying flooding conditions during other stages of growth [12, 13, 16]. There have been relatively few studies on the photosynthesis of rice flag leaves during the grain-filling stage that have used plants grown under nonflooded cultivation for the whole growth period. It is very interesting to know photosynthetic characteristics and photosynthetic production capacity during the grain-filling stage under long-term nonflooded cultivation. Such research will also contribute to an understanding of the rice grain yield production potential under long-term nonflooded cultivation.

Light energy is a driving factor of photosynthesis. Light energy absorbed by chlorophyll molecules in a leaf can be lost from photosynthesis (photochemistry), heat loss, and chlorophyll fluorescence [17]. The processes of photochemistry, chlorophyll fluorescence, and heat loss directly compete for excitation energy [18]. Therefore, chlorophyll fluorescence, especially for the PSII system, can be a very powerful tool to study photosynthetic performance, the degree of water stress or photoinhibition, and photoprotective mechanisms [17–19]. Under abiotic stress, the photosynthetic rate is significantly reduced [12], and the requirement for photosynthetic electrons also decreases so that surplus radiant energy must be degraded in alternative ways [20]. To alleviate or avoid damaging the plant's photosynthetic apparatus, excessive energy can be dissipated by heat emission (nonphotochemical quenching); this mechanism protects the photosynthetic apparatus and helps to resist photoinhibition [21]. Photoinhibition occurs when there is a large surplus of radiant energy and/or nonphotochemical quenching is insufficient to dissipate excess energy [22]. To date, there has been little systematic research on the photosynthetic attributes (light energy absorption, proportion of energy distributed to the photosynthetic apparatus, photoprotection, and adaptation mechanisms) of rice grown under long-term nonflooded irrigation.

In rice, photosynthesis is affected by nonflooded cultivation conditions. The photosynthetic capacity is significantly improved in the mulching cultivation than in the bare land cultivation [7]. It is likely that different nonflooded cultivation methods will affect photosynthetic performance, photoprotection, and adaptation mechanisms, ultimately affecting yield. Therefore, in this study, we cultivated rice using three different nonflooded cultivation methods and analyzed various photosynthetic parameters. The objectives of this study are (1) to characterize photosynthesis, photoprotection, and adaptation mechanism of flag leaves during grain-filling under long-term nonflooded irrigation management and (2) to evaluate the photosynthetic production potential under different cultivation conditions.

## 2. Materials and Methods

### 2.1. Plant Material and Experiment Design

**Figure 1:**
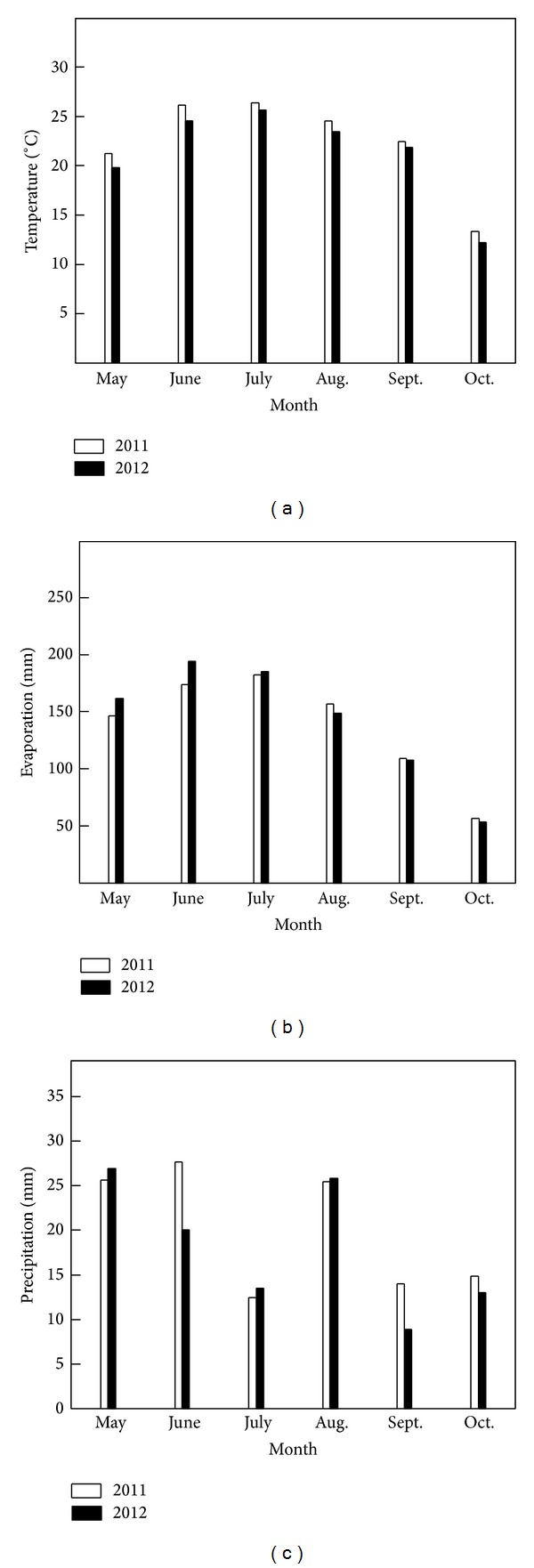
Average temperature (a), evaporation (b), and precipitation (c) during the rice growing period in Shihezi in 2011 and 2012. Each datum point represents the mean of a month.

Field experiments using the rice (*Oryza sativa* L.) cultivar Ninggeng28 were conducted in 2011 and 2012 at the Agricultural Drought Research Institute of the TianYe Group Company (44°26.5′N, 86°01′E), Xinjiang Province, China. The average temperature, evaporation, and precipitation of the experimental sites were 21.14–22.41°C, 825.30–851.40 mm, and 108.10–120.01 mm, respectively, during the rice growing season (May to October) in both years (Figures 1(a), 1(b), and 1(c)). The value of photosynthetically active radiation during experimental time (10:30–12:30) was 1000–1250 *μ*mol photon m^−2^ s^−1^ in both years. The soil properties of the experimental sites were similar and its chemical and physical properties listed in Table 1.

**Figure 2:**
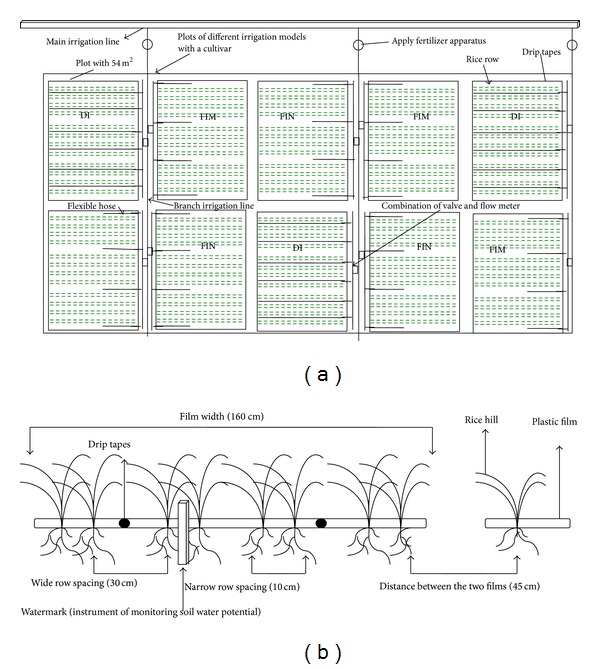
Experiment layout (a) and sketch map of planting mode (b) under nonflooded irrigation treatments in Shihezi in both years.

**Figure 3:**
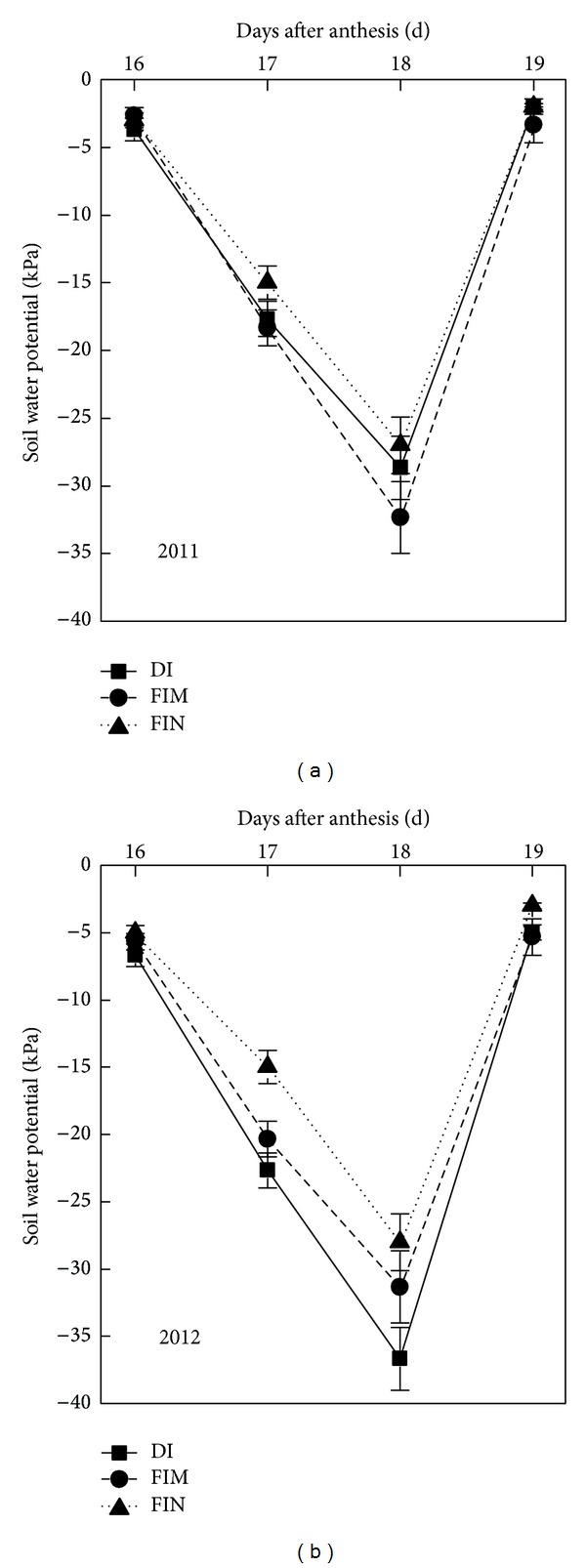
Soil water potential at 0–20 cm soil layer under nonflooded irrigation treatments from the 16th day after anthesis to the 19th day after anthesis in Shihezi in 2011 (a) and 2012 (b). Vertical bars represent SE of the mean; the SE was calculated across three replicates for each year.

The nonflooded irrigation treatments (DI, FIM, and FIN treatments) were sown by artificial hand dibbling at a depth of 3 cm on April 18 in 2011 and April 23 in 2012, and then irrigated at a rate of 450 m^3^ ha^−1^ on the next day after sowing to ensure normal germination because of the dry direct-seeded system used in this study. No further irrigation was supplied until the third leaf emerged. The three nonflooded irrigation treatments were arranged in a completely randomized design with three replicates (Figure 2(a)). The size of each plot was 5.5 m × 10 m. Waterproof membranes were buried to a depth of 60 cm below the soil surface between adjacent plots in the nonflooded irrigation treatments to prevent water exchange. All plots were covered by plastic film before sowing. Two drip tapes (emitter discharge rate, 3.20 L h^−1^; emitter spacing, 0.30 m) were laid under the plastic film for the DI treatment. Flexible hoses with a 2 cm diameter were located on both sides of the plastic film to supply water in the FIM and FIN treatments (Figure 2(a)). In the FIM and FIN treatments, 20 cm high dams were constructed and covered with plastic film to minimize water surface runoff. Holes were opened on the membrane surface when the rice was sown. The planting density was 45.71 hills m^−2^ with a 10–30-10–30-10–30-10–45 cm row-spacing configuration. The plant spacing within the hills was 10 cm (Figure 2). The planting density were mainly based on the results of a preliminary study about directly seeded rice under drip irrigation with full mechanization in recent years. Basic seedling numbers per hill were six plants in both years. The water regimes were applied from the 3-leaf stage to maturity. Forty to sixty millimeter of water was applied when the soil water potential in the 0–20 cm soil layer of the narrow rows (Figure 2(b)) dropped to −30 KPa, as monitored by tensiometer (Watermark, Irrometer Company, Riverside, CA). This soil water potential threshold was established based on the results of a preliminary field investigation in 2010. The soil water potential at the 20 cm depth occasionally dropped to −40 KPa during the rice growing period. To create the bare-soil treatment (i.e., the FIN treatment), the plastic films were removed at the 3-leaf stage. The hydrological conditions during the data acquisition period are shown in Figure 3. When the threshold values for supplementary irrigation were reached, supplementary irrigation was immediately applied. In particular, plants grown in nonflooded irrigation treatments were irrigated after acquiring gas exchange and chlorophyll fluorescence parameters during the data acquisition period. The CF cultivation treatment was set as CK; the planting density of the CF treatment was 40 hills m^−2^ (10 cm × 25 cm) with five plants per hill. The seeds were sown on the nursery trays on the same day when the nonflooded irrigations were sown, and then twenty-one-day-old seedlings from the nursery trays were transplanted. A water depth of 5–10 cm was continuously maintained in the CF treatment from transplanting to maturity. The sites of the CF treatment were about 150 m away from the nonflooded irrigation plots and the soil physicochemical property was consistent with nonflooding irrigation treatments in both years (the flooding treatment was set in three small pools built in 2010, and the flooding plots were not previously traditional paddy; the area of the each small pool was also the same with the plot of nonflooding irrigation treatments).

Fertilizers applied were 270 kg N per hectare as urea, 100 kg K_2_O per hectare as potassium chloride, 90 kg P_2_O_5_ per hectare as calcium superphosphate, and 30 kg zinc sulfate per hectare, of which 10% N, all K_2_O, all P_2_O_5_, and all Zn were applied as basal fertilizer; the rest of N was applied in four split applications: 20% at the three leaf blades stage, 35% at tillering, 35% at panicle initiation, and 10% at flowering.

### 2.2. Measurement of Dry Matter Preanthesis (DMPrA), Dry Matter Postanthesis (DMPA), Leaf Area Index at Flowering (LAI_max_), Matter Translocation, and Grain Yield

To measure DMPrA, DMPA, and (LAI_max⁡_) (green leaf area at flowering), 0.50 m^2^ plant samples were taken from each plot at the flowering period (August 2 in 2011 and August 4 in 2012) and the mature stage (September 13 in 2011 and September 12 in 2012) in the two years. The samples were separated into stem, leaf, and panicle when present. The leaf area was measured with a LI-3100 leaf area meter (LI-COR Inc., Lincoln, NE,USA). Then samples were dried in an oven at 75°C for 72 h and the weights of stem, leaf, and panicle were measured. The dry matter at the flowering period was defined as DMPrA. DMPA was calculated as follows: dry matter at maturity—dry matter at anthesis. Matter translocation = dry weight of stems and sheaths at anthesis—dry weight of stems and sheaths at maturity. The parameter of matter translocation was measured because the stems and sheaths are considered as important energy storage organs and the matter translocation rate will affect grain yield formation [8, 9]. At maturity (September 15, 2011 and September 14, 2012), 8 m^2^ plants in each plot were harvested to calculate grain yield. Grain weight is expressed at 14% moisture content.

### 2.3. Measurement of Chlorophyll Content

Fresh flag leaf tissue (0.1 g) was extracted in 10 mL of 80% (v/v) acetone under dark and sealed conditions for 48 h. The absorbance (OD values) of the solution was measured with a 722S visible spectrophotometer (Shanghai Third instrument Factory, Shanghai, China) at 647 nm and 664 nm. In case of highly elevated titers (optical density [OD] values > 0.8), the extracted solution was further diluted in 80% (v/v) acetone to give OD values of between 0.1 and 0.8. The chlorophyll content was calculated as described by Porra [23], as follows: Chl *a* (mg L^−1^) = [12.25 × OD_664_  − 2.55 × OD_647_] × dilution ratio. Chl *b* (mg L^−1^) = [20.31 × OD_647_  − 4.91 × OD_664_] × dilution ratio. Chl (*a* + *b*) (mg L^−1^) = [17.76 × OD_647_ + 7.34 × OD_664_] × dilution ratio. Chl *a/b* = Chl *a*/Chl *b*. Finally, Chl (mg g^−1^ FW) = Chl (mg L^−1^) × 0.01 L/0.1 g FW (fresh weight).

### 2.4. Measurement of Total Nitrogen Content (TNC) and Nitrogen Content per Leaf Area (NLA) of Flag Leaves

To measure total nitrogen content, flag leaves were harvested on the 16th day after anthesis in 2012. The TNC of the dried tissues was determined using the micro-Kjeldahl method [24]. The amount of nitrogen per leaf area (NLA) was calculated by dividing TNC by the specific leaf weight. The specific leaf weight of the flag leaf was defined as flag leaf weight per unit area.

### 2.5. Measurement of Root Activity

The root activity was determined on the 20th day after anthesis in 2012 using the triphenyltetrazolium chloride (TTC) method [25]. Fresh and young roots from the 0–20 cm soil layer (which contained approx. 85% of the root system; data not presented) were washed and cut at 2 cm from the root tips. Then, approximately 0.30 g fresh root tissue was placed into test tube with 5 mL of 0.40% (w/v) TTC and 5 mL phosphate buffer (0.06 mol L^−1^, pH = 7). Tubes were incubated at 37°C in a water bath for 3 h, and then 2 mL of 1 mol L^−1^ sulfuric acid was added to each tube to stop the reaction. The roots were then picked out and ground in a pestle with 3-4 mL of ethyl acetate and a little quartz sand. The liquid phase was removed into a test tube and the pestle was washed 2-3 times with a small volume of ethyl acetate. The absorbance (OD values) of the extractants was recorded at 485 nm with a 722S visible spectrophotometer (Shanghai Third instrument Factory, Shanghai, China).

### 2.6. Measurement of Net Photosynthetic Rate (*P*_*N*_), Stomatal Conductance (*g*_*s*_), Intercellular CO_2_ Concentration (*C*_*i*_), and Transpiration Rate (*E*)

Five individual flag leaves in vivo were sampled and labeled for each plot to acquire gas exchange parameters and chl *a* fluorescence across treatments and years. Gas exchange parameters were measured with a photosynthesis system (Li-6400, LI-COR Biosciences, Lincoln, NE, USA) at 10:30–12:30 h from the 16th day after anthesis to the 19th day after anthesis under 1200 *μ*mol photon m^−2^ s^−1^ light intensity. The light was provided by a red/blue LED light source system in both years. The gas exchange parameters including *P*_*N*_ (*μ*mol m^−2^ s^−1^), *g*_*s*_ (mol m^−2^ s^−1^), *C*_*i*_ (*μ*mol mol^−1^), and *E* (mmol m^−2^ s^−1^) were acquired. The atmospheric CO_2_ concentration, air temperature, and relative air humidity were 380–390 *μ*mol mol^−1^, 26–28°C, and 60.21–65.10%, respectively, during the data acquisition period of both study years.

### 2.7. Measurement of Chlorophyll Fluorescence Analysis

Chl *a* fluorescence was synchronously measured with gas exchange at 10:30–12:30 h by a portable saturation pulse fluorometer (PAM-2100, Walz GmbH, Effeltrich, Germany), equipped with a 2030-B leaf clip holder, which can monitor PAR and leaf temperature simultaneously. The leaves were continuously illuminated at 1200 *μ*mol m^−2^ s^−1^ actinic light with 5 minutes to measure the steady-state fluorescence yield (*F*_*s*_), then the maximal fluorescence level (*F*_*m*_′) in the light-adapted leaves were recorded after a 0.80-s saturating pulse (8000 *μ*mol m^−2^ s^−1^). Meanwhile, maximal fluorescence yield of dark-adapted state (*F*_*m*_) and minimum fluorescence yield of dark-adapted state (*F*_*o*_) were measured at predawn, being also with the labeled flag leaves (Note that leaf temperatures monitored by a 2030-B leaf clip holder were 19.6–21.8°C at this time across both years). *F*_*o*_ was determined under illumination with far-red light (<1 *μ*mol m^−2^ s^−1^), and then a 0.80-s saturating pulse (8000 *μ*mol m^−2^ s^−1^) was supplied to determine *F*_*m*_. The minimum fluorescence yield in light-adapted state (*F*_*o*_′) was calculated as *F*_*o*_′ = *F*_*o*_/[(*F*_*v*_/*F*_*m*_)+(*F*_*o*_/*F*_*m*_′)] [26]. The potential maximum efficiency of PSII was estimated as *F*_*v*_/*F*_*m*_ = (*F*_*m*_ − *F*_*o*_)/*F*_*m*_. The PSII maximum efficiency was estimated as *F*_*v*_′/*F*_*m*_′ = (*F*_*m*_′ − *F*_*o*_′)/*F*_*m*_′ [27]. The actual PSII efficiency of PSII was estimated as Φ_PSII_ = (*F*_*m*_′ − *F*_*s*_)/*F*_*m*_′ [27]. Nonphotochemical quenching was estimated as NPQ = (*F*_*m*_ − *F*_*m*_′)/*F*_*m*_′ [28]. The photochemical quenching coefficient which also represents the fraction of open PSII reaction centers was estimated as *q*_*p*_ = (*F*_*m*_′ − *F*_*s*_)/(*F*_*m*_′ − *F*_*o*_′) [29].

### 2.8. Statistical Analysis

Data were analyzed using the generalized linear model (GLM) procedure (SPSS16.0, SPSS Inc., Chicago, USA). The effects of the various factors included cultivation mode, year, and interactions of cultivation mode × year were assessed by analysis of variance. Means were compared by Fisher's least-significant-difference test at the 5% probability level. Also, two-tailed *t*-test was used to compare with the difference of gas exchange and chlorophyll fluorescence parameters gas exchange and between 16th day after anthesis and 19th day after anthesis.

## 3. Results

### 3.1. Analysis-of-Variance of Treatments

As shown in Table 2, the DMPA, grain yield, Chl, *P*_*N*_, *g*_*s*_, *C*_*i*_, *F*_*v*_/*F*_*m*_, *F*_*v*_′/*F*_*m*_′, Φ_PSII_, and NPQ were affected significantly by the different cultivation modes (*P* < 0.001). However, there were no significant differences between the two years for all parameters (*E*, *q*_*P*_, and DMPrA not present). There was a significant interaction effect between the cultivation mode and year for *F*_*v*_′/*F*_*m*_′ (*P* < 0.05), but not for any of the other parameters. Therefore, data from two study years were averaged for further analyses.

### 3.2. DMPrA, DMPA, LAI_max_, Matter Translocation, and Grain Yield

In plants grown under nonflooded irrigation, the DMPrA was slightly higher and the DMPA was significantly lower than that of plants in the CF treatment (*P* < 0.05; Table 3). The DI treatment had the highest leaf area index at flowering stage, followed by the FIM treatment, the FIN treatment, and then the CF treatment (Table 3). The matter translocation was in the order of CF > DI > FIM > FIN treatments (Table 3). As Table 3 showed, the grain yield was about 8500 kg ha^−1^ in the CF treatment and ranged from 2100 kg ha^−1^ to 5800 kg ha^−1^ in the nonflooded irrigation treatments. The grain yield was significantly higher in the DI treatment than in the FIM and FIN treatments (*P* < 0.05; Table 3).

### 3.3. Changes in Chlorophyll Content, Root Activity, TNC, and NLA

**Figure 4:**
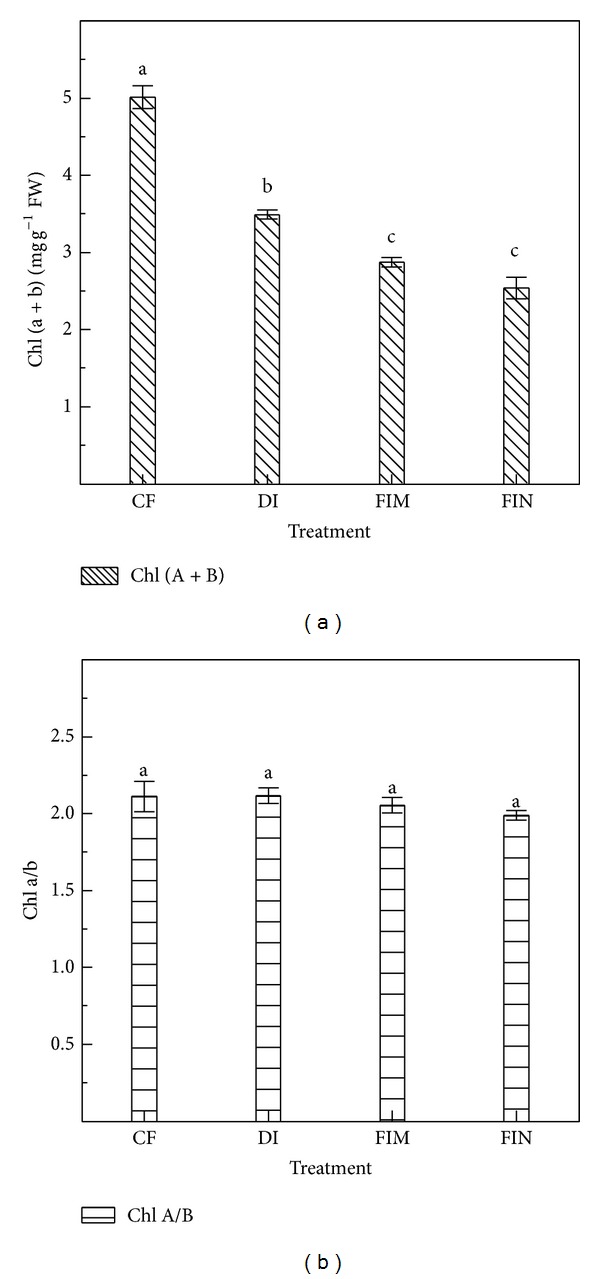
Chl content of cultivar Ninggeng28 (*Oryza sativa* L.) under CF, DI, FIM, and FIN treatments at the grain-filling stage, respectively, in Shihezi. The CF indicates conventional flooding, and the DI, FIM, and FIN are plastic film mulching with drip irrigation, plastic film mulching with furrow irrigation, and no mulching with furrow irrigation, respectively. Vertical bars represent SE of the mean; the SE was calculated across five replicates for each year and averaged for the 2 years. Values of different treatments followed by the same letter indicate no significant differences (*P* < 0.05) according to least-significant-difference test.

The Chl (*a* + *b*) and Chl *a*/*b* values determined on the 4 days of measurement (from the 16th day after anthesis to the 19th day after anthesis) were averaged for the same treatment, because no significant differences were observed among the different days of the data acquisition period (data of analysis-of-variance not present). The results showed that the Chl (*a* + *b*) concentration in the CF treatment (control) was 5.01 mg g^−1^ FW, which was 53.10% higher than in the DI treatment, 69.12% higher than in the FIM treatment, and 73.31% higher than in the FIN treatment (Figure 4(a)). No significant differences were observed in Chl *a/b* among the four treatments (*P* > 0.05; Figure 4(b)).

At the grain-filling stage, the root activity in the CF treatment was the highest, which was significantly increased by 21.93% compared with the DI treatment, by 38.39% compared with the FIM treatment, and by 50.64% compared with the FIN treatment (*P* < 0.05; Table 3). The highest TNC and NLA values for the flag leaves were in the CF treatment, followed by the DI treatment, then the FIM treatment, and then the FIN treatment. Significant differences in the TNC and NLA parameters existed among the four treatments (*P* < 0.05; Table 3).

### 3.4. Analysis of Leaf Gas Exchange

**Figure 5:**
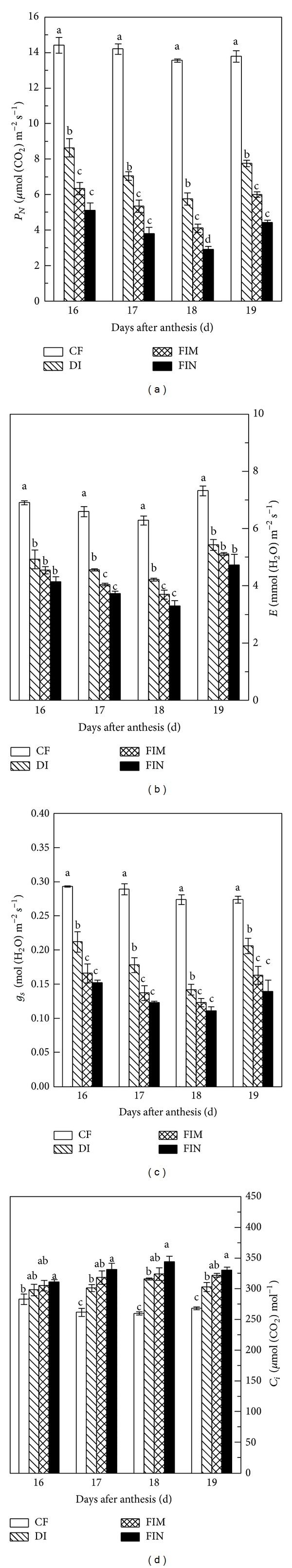
The *P*_*N*_ (a), *E* (b), *g*_*s*_ (c), and *C*_*i*_ (d) of cultivar Ninggeng28 (*Oryza sativa* L.)under different treatments from the 16th day after anthesis to the 19th day after anthesis in Shihezi. The CF indicates conventional flooding, and the DI, FIM, and FIN are plastic film mulching with drip irrigation, plastic film mulching with furrow irrigation, and no mulching with furrow irrigation, respectively. Vertical bars represent SE of the mean, the SE was calculated across five replicates for each year and averaged for the 2 years. Values of each measurement date followed by the same letter indicate no significant differences (*P* < 0.05) according to least-significant-difference test.

Plants in the DI treatment had a higher *P*_*N*_ than did plants in the FIM and FIN treatments from the 16th day after anthesis to the 19th day after anthesis. However, the *P*_*N*_ of plants in the DI, FIM, and FIN treatments was always significantly lower than that of plants in the CF treatment during the data acquisition period (Figure 5(a)). The *P*_*N*_ in the CF treatment remained relatively stable from the 16th day after anthesis to the 19th day after anthesis (Figure 5(a)). In contrast, plants grown in the nonflooded irrigation treatments showed decreasing *P*_*N*_ as the soil water potential decreased, with minimum *P*_*N*_ values on the 18th day after anthesis (Figure 5(a)), which the soil water potential on the day reached its lowest values across treatments and years (from −28 KPa to −36.50 KPa) (Figures 3(a) and 3(b)). After that, the *P*_*N*_ rose on the 19th day after anthesis, when the soil water potential had reached −3 and −5.40 KPa as a result of supplementary irrigation in the DI and FIM treatments, respectively. No significant difference were observed for the parameter between the 16th day after anthesis and the 19th day after anthesis in the DI and FIM treatments, but significant difference existed in the FIN treatment (two-tailed *t*-test. For the DI treatment: df = 5, *t* = 1.08, *P* = 0.31; for the FIM treatment: df = 5, *t* = 1.38, *P* = 0.20; for the FIN treatment: df = 5, *t* = 4.31, *P* = 0.01), indicating that the *P*_*N*_ could completely recover after supplementary irrigation in the DI and FIM treatments. The trends in *g*_*s*_ and *E* values were similar to those observed for *P*_*N*_ across soil water potential and cultivation modes. Their values were significantly lower under the nonflooded irrigation treatments than under the CF treatment (*P* < 0.05). Meanwhile, both *g*_*s*_ and *E* parameters gradually decreased as the soil water potential decreased until the 18th day after anthesis in the nonflooded irrigation treatments, and then recovered on the 19th day after anthesis, after supplementary irrigation, in the DI and FIM treatments compared with the 16th day after anthesis (two-tailed *t*-test; *P* > 0.05 for the FIM and DI treatments; *P* < 0.05 for the FIN treatment. Data of analysis-of-variance not present). The variation in *C*_*i*_ was small among the different treatments, but higher *C*_*i*_ values were observed in the nonflooded irrigation treatments than in the CF treatment during the entire measurement period (Figure 5(d)).

### 3.5. Analysis of Chlorophyll Fluorescence

**Figure 6:**
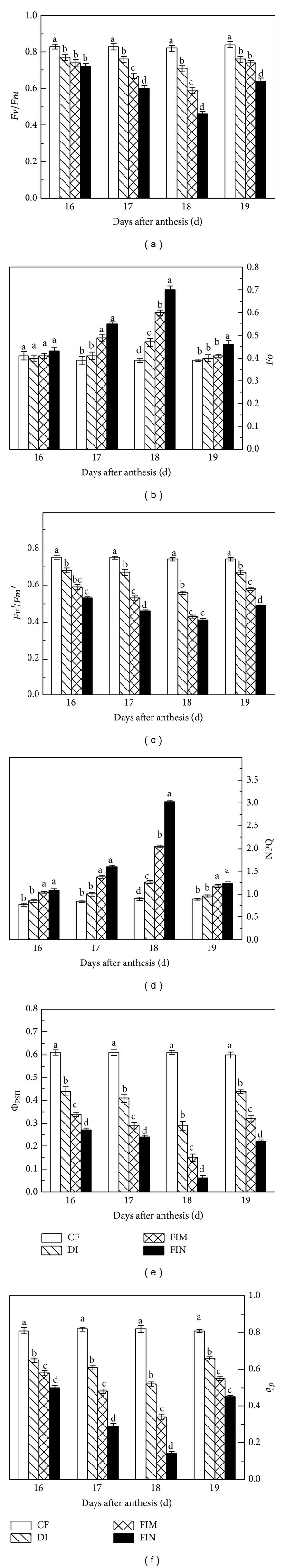
The *F*_*v*_/*F*_*m*_ (a), Fo (b), *F*_*v*_′/*F*_*m*_′ (c), NPQ (d), Φ_PSII_ (e), and *q*_*p*_ (f) of cultivar Ninggeng28 (*Oryza sativa* L.) under different treatments from the 16th day after anthesis to the 19th day after anthesis in Shihezi. The CF indicates conventional flooding, and the DI, FIM, and FIN are plastic film mulching with drip irrigation, plastic film mulching with furrow irrigation, and no mulching with furrow irrigation, respectively. Vertical bars represent SE of the mean; the SE was calculated across five replicates for each year and averaged for the 2 years. Values of each measurement date followed by the same letter indicate no significant differences (*P* < 0.05) according to least-significant-difference test.

The values of *F*_*v*_/*F*_*m*_, *F*_*v*_′/*F*_*m*_′, *q*_*p*_, and Φ_PSII_ in the nonflooded irrigation treatments were significantly lower than their respective values in the CF treatment from the 16th day after anthesis to the 19th day after anthesis (*P* < 0.05; Figures 6(a), 6(c), 6(e), and 6(f)). During the four days, both NPQ and Fo values in the nonflooded irrigation treatments were significantly higher than in the CF treatment (*P* < 0.05; Figures 6(b) and 6(d)). For the CF treatment, all fluorescence parameters were relatively stable. However, for the nonflooded irrigation treatments, values of the *F*_*v*_/*F*_*m*_, *F*_*v*_′/*F*_*m*_′, *q*_*p*_, and Φ_PSII_ decreased with decreasing soil water potential until the 18th day after anthesis, and then completely recovered after supplementary irrigation on the 19th day after anthesis in the DI and FIM treatments. These results were supported by two-tailed *t*-test, which showed no significant differences between the 16th day after anthesis and the 19th day after anthesis in the DI and FIM treatments (*P* > 0.05 in the FIM and DI treatments; *P* < 0.05 in the FIN treatment. Data of analysis-of-variance not present). Under nonflooded irrigation, *F*_*v*_/*F*_*m*_, *F*_*v*_′/*F*_*m*_′, *q*_*p*_, and Φ_PSII_ were always significantly higher in the DI treatment than in the FIM and FIN treatments during data observation period (*P* < 0.05; Figure 6). The *F*_*o*_ and NPQ values were significantly higher under the nonflooded irrigation treatments than under the CF treatment, and gradually increased as the water potential decreased until the 18th day after anthesis. Then, both *F*_*o*_ and NPQ values substantially decreased on the 19th day after anthesis because of supplementary irrigation (Figures 6(b) and 6(d)).

## 4. Discussion

Dry matter accumulation is a critical factor in grain yield formation. The aboveground biomass before anthesis may contribute 20–40% to the final crop yield [30], and the rest of the matter that contributed to grain yield is derived from photosynthesis during the grain-filling stage [8, 9]. In our study, there were no significant differences in the DMPrA among treatments. However, the matter translocation in the FIM and FIN treatments was significantly lower than that in the CF and DI treatments (*P* < 0.05; Table 3). The low translocation efficiency may have been insufficient to achieve a high grain yield in the FIM and FIN treatments [9]. For the DI treatment, the matter translocation was only slightly reduced compared with the CF treatment. Therefore, we infer that the decreased grain yield could mainly depend on the photosynthetic capacity during the grain-filling stage in the DI treatment (Figure 5(a)) and also be related to the dry matter translocation efficiency at the preanthesis stage in the FIM and FIN treatments (Figure 5(a); Table 3).

Moreover, a significantly higher population structure (LAI_max⁡_) was observed in nonflooded irrigation treatments than in the CF treatment (*P* < 0.05; Table 3). We can speculate that the high LAI_max⁡_ negatively affected biomass accumulation and yield formation under nonflooded irrigation. There are two main reasons for this speculation: first, a considerable proportion of the crop was invalid tillers (nonproductive tillers), especially in the FIM and FIN treatments (data not shown). The accumulated assimilates are rarely transferred from invalid tillers to effective panicles, and this large proportion of redundant matter and energy is greatly wasted in poorly performing tillers. Thus, grain yield formation could be limited by inadequate material supply [31]. Second, the consumption of photosynthate would increase because the respiration rate of C_3_ plants increases almost proportionally to the increase in LAI [32]. Finally, crop failure could also be relevant to the poor population structure.

The *P*_*N*_ in the FIN treatment was significantly lower than that in the CF treatment. This result was supported by previous studies [7]. Plants in the treatments with plastic mulch (DI and FIM treatments) showed lower *P*_*N*_ than those in the CF treatment, which differs from the result found by Zhang et al. [7]. In the nonflooded irrigation treatments, Chl (*a* + *b*) showed significant declines compared with the CF treatment (*P* < 0.05; Figure 4). Thus, low *P*_*N*_ in graminaceous crops such as maize (*Zea mays* L.) and rice (*Oryza sativa* L.) is apparently associated with lower levels of quantity of pigments [33, 34].

Nitrogen plays an important role during photosynthesis, since the proteins of the Calvin cycle and thylakoids represent the majority of leaf nitrogen. Photosynthesis of rice leaves significantly increases with increasing nitrogen content of leaves [35, 36]. Mae [36] showed that the maximum rate of CO_2_ assimilation per unit leaf area (measured under light-saturated, ambient air condition) was almost proportional to the NLA. Therefore, the photosynthetic capacity of leaves is closely related to the nitrogen content [37]. In our studies, the TNC and NLA in the CF treatment were significantly higher than that in the nonflooded irrigation treatments (*P* < 0.05; Table 3). The results suggest that the underlying reason which limited photosynthetic capacity was mainly the lower nitrogen content in the nonflooded irrigation treatments, compared with that in the CF treatment. Although the bad performance of the root activity could be one of the important reasons that restricted the absorption of nitrogen [38], it is still very necessary to conduct the future experiment to find out the scientific sound reason for the low nitrogen content in the nonflooded irrigation.

Jones [39] suggested that the values of *C*_*i*_ and *P*_*N*_ can be a useful criterion to determine whether photosynthesis is limited by stomatal closure or metabolic impairment. Compared with normal (control) treatment, a decrease in *P*_*N*_ alongside a decrease in *C*_*i*_ indicates stomatal limitation of photosynthesis, whereas if the *P*_*N*_ decreases while *C*_*i*_ increases, then nonstomatal limitation (metabolic impairment) of photosynthesis is predominant [40]. Under the nonflooded irrigation treatments, the *C*_*i*_ was also higher than in the CF treatment during the data acquisition period (Figure 5(d)), but their *P*_*N*_ values were significantly lower than those in the CF treatment (*P* < 0.05; Figure 5(a)). In addition, the *g*_*s*_ values ranged from 0.12 to 0.23 mol m^−2^ s^−1^ under the nonflooded irrigation treatments, which was significantly lower than in the CF treatment (*P* < 0.05; Figure 5(c)). The low *g*_*s*_ would probably decrease the levels of ribulouse bisphosphate, impair ATP synthesis, and even inhibit Rubico activity [40], which together would inhibit *P*_*N*_.

Under the nonflooded irrigation treatments, both *P*_*N*_ and *g*_*s*_ declined with soil water potential decrease. The *P*_*N*_ and *g*_*s*_ almost completely recovered after supplementary irrigation in the plastic mulching treatments (DI and FIM treatments) but not in the FIN treatment (Figures 5(a) and 5(c)). This indicated that photoinhibition might not be permanent in the DI and FIM treatments; that is, it could recover during the grain-filling stage if the soil water potential remained between 0 and −30 KPa during the entire growth period. Moreover, the values of *P*_*N*_, *g*_*s*_, and *E* were higher under the DI treatment than under the FIM and FIN treatments (Figures 5(a), 5(c), and 5(d)). The results show that the DI treatment can significantly improve rice photosynthetic performance under nonflooded irrigation.

The use of chlorophyll fluorescence to monitor photosynthetic performance in plants is now widespread. Chl *a* fluorescence is a very sensitive tool to study the stress-induced damage to photosystem 2 (PSII) [41]. The *F*_*v*_/*F*_*m*_ value is frequently used as an indicator of stresses caused to PSII [18, 42]. Under the nonflooded irrigation treatments, the *F*_*v*_/*F*_*m*_ values were significantly lower than that in the CF treatment which its value was 0.81–0.83 during data acquisition period and fell sharply as the soil water potential reduced from the 16th day after anthesis to the 18th day after anthesis (Figure 6(a)). This result indicated that the rice crop could be subject to water stress during grain-filling stage. Also, increasing of *F*_*o*_ value is usually considered as an important parameter to estimate photoinhibition in many plants [19, 43]. Our results showed that the *F*_*o*_ sharply increased when soil water potential dropped to −15 KPa in the FIN treatment, −20 KPa in the FIM treatment, and −36.50 KPa in the DI treatment, compared with that in the CF treatment, and then recovered when soil water potential returned to −4.50 KPa after supplementary irrigation for the DI and FIM treatments (Figure 6(b)). These results imply that nonflooded irrigation cultivation could cause rice to suffer from photoinhibition when the soil water potential ranges from −15 KPa to −36.50 KPa. The plants in the DI treatment were more tolerant to nonflooded irrigation cultivation practices and showed higher photosynthetic efficiency than that in FIM and FIN treatments.

The Φ_PSII_ reflects the efficiency of excitation energy captured by open reaction centers of PSII. Two factors contribute to Φ_PSII_ [27]: one being the fraction of PSII centers in the open state *Q*_*A*_ (primary quinone acceptor of PSII), for which the quantitative value is equivalent to *q*_*p*_ [18], and the other being *F*_*v*_′/*F*_*m*_′ [27]. Our studies suggested that the decreases in Φ_PSII_ under nonflooded irrigation were caused by a combination of two processes (Figures 6(c), 6(e), and 6(f)). Previous studies indicated that a decrease in *q*_*p*_ could lead to an increase in the levels of toxic singlet oxygen form [44, 45]. Unfortunately, we did not quantify reactive oxygen species (ROS) in the present study. Further research is required to determine whether ROS are produced under nonflooded conditions and to examine their scavenging mechanisms. In addition, the decrease in *F*_*v*_′/*F*_*m*_′ is considered to reflect a proactive and photoprotective thermal dissipation process in which excess excitation energy is depleted before it reaches the PSII centers [45]. Figure 6 shows that the *F*_*v*_′/*F*_*m*_′ values significantly decreased under nonflooded irrigation treatments and greatly decreased as the soil water potential decreased. These results indicated that thermal dissipation processes were enhanced before excess energy reached the PSII centers under nonflooded irrigation, especially when the soil water potential was low, and in the FIN treatment. In brief, under nonflooded irrigation treatments, total thermal dissipation, including depletion of heat by NPQ (Figure 6(d)) and by the processes reflected by the *F*_*v*_′/*F*_*m*_′ value (Figure 6(c)) pathways, was significantly higher than that under the CF treatment during the whole observation period. These pathways could be important photoprotective mechanisms in rice plants cultivated under nonflooded irrigation conditions.

## 5. Conclusions

Under nonflooded treatments, photosynthetic capacity and grain-yield of rice plants were significantly decrease compared with the CF treatment. Nonphotochemical quenching (NPQ) played an important role in protecting photosynthetic apparatus in flag leaf of rice plants against damage by nonflooded environments. To improve photosynthetic productivity in nonflooded irrigation, it could be necessary to promote nitrogen content in flag leaf. Also, the reasons in regulating nitrogen metabolism in flag leaf are not clear in nonflooded irrigation, and it need to do follow-up studies to find out scientific mechanisms.

## Figures and Tables

**Table 1:** Soil characteristics of the field experiments conducted in Shihezi in 2011 and 2012. Values are the mean ± standard error (SE); the SE was calculated across five replicates in both years and averaged for the nonflooded irrigation and conventional flooding sites.

Parameter	2011	2012
Clay (%)	23 ± 3.32	19 ± 0.98
Silt (%)	38 ± 2.31	34 ± 1.43
Sand (%)	43 ± 3.19	41 ± 2.26
pH	7.51 ± 0.32	7.72 ± 0.18
Olsen-P (mg kg^−1^)	28.46 ± 4.24	22.13 ± 1.33
Organic matter (mg kg^−1^)	25.46 ± 0.95	26.35 ± 1.11
Alkaline-N (mg kg^−1^)	60.83 ± 2.49	58.72 ± 1.65
Available potassium (mg kg^−1^)	342.54 ± 54.13	313.42 ± 32.17
Soil saturation volume moisture content (%)	30.11 ± 0.12	32.57 ± 0.25

**Table 2:** Analysis-of-variance (*F*-values) for chlorophyll (Chl), net photosynthetic rate (*P*_*N*_), stomatal conductance (*g*_*s*_), intercellular CO_2_ concentration (*C*_*i*_), maximal quantum yield of PSII photochemistry (*F*_*v*_/*F*_*m*_), PSII maximum efficiency (*F*_*v*_′/*F*_*m*_′), effective quantum yield of PSII photochemistry (Φ_PSII_), and nonphotochemical quenching (NPQ) between/among years and treatments.

Source of variation	df	DMPA	Yield	Chl	*P*_*N*_	*g*_*s*_	*C*_*i*_	*F*_*v*_/*F*_*m*_	*F*_*v*_′/*F*_*m*_′	Φ_PSII_	NPQ
Cultivation mode	3	421.55***	351.21***	163.10***	141.22***	13.73***	20.91***	45. 72***	143.24***	178.31***	16.43***
Year	1	4.15^ns^	3.78^ns^	2.27^ns^	4.25^ns^	0.03^ns^	0.26^ns^	2.28^ns^	2.65^ns^	0.89^ns^	0.07^ns^
C × Y	3	3.11^ns^	2.41^ns^	1.05^ns^	0.32^ns^	0.57^ns^	0.47^ns^	2.94^ns^	3.56*	1.17^ns^	0.08^ns^

*Significance difference at *P* < 0.05.

***Significance difference at *P* < 0.001.

^ns^Nonsignificant differences.

**Table 3:** The dry matter preanthesis (DMPrA), dry matter postanthesis (DMPA), matter translocation, grain yield, leaf area index at flowering stage (LAI_max_), root activity, total nitrogen content (TNC), and nitrogen content per leaf area (NLA) of cultivar Ninggeng28 (*Oryza sativa* L.) under different treatments in Shihezi.

	DMPrA kg ha^−1^	DMPA kg ha^−1^	Matter translocation kg ha^−1^	Yield kg ha^−1^	LAI_max_	Root activity *μ*g g^−1^ FW h^−1^	TNC *μ*g g^−1^	NLA *μ*g dm^−2^
CF	10537.22 ± 265.36^b^	15262.93 ± 473.01^a^	1384.65 ± 73.24^a^	8579.85 ± 110.43^a^	5.49 ± 0.49^c^	524.43 ± 40.33^a^	8.37 ± 0.51^a^	3.71 ± 0.22^a^
DI	13398.54 ± 511.23^a^	9417.22 ± 303.75^b^	1362.31 ± 89.18^a^	5793.68 ± 238.41^b^	7.16 ± 0.28^a^	409.43 ± 10.40^b^	6.21 ± 0.13^b^	2.19 ± 0.13^b^
FIM	11845.43 ± 368.24^ab^	5881.14 ± 569.50^c^	450.37 ± 55.41^b^	3636.92 ± 154.64^c^	6.77 ± 0.28^ab^	323.09 ± 19.91^c^	4.09 ± 0.43^c^	1.13 ± 0.15^c^
FIN	11862.40 ± 821.14^ab^	4666.02 ± 118.45^d^	−324.55 ± 63.59^c^	2086.01 ± 221.41^d^	6.64 ± 0.14^b^	258.86 ± 32.50^d^	3.64 ± 0.12^c^	0.92 ± 0.08^d^

Values are the mean ± SE; the SE was calculated across three replicates for each year and averaged for the 2 years. Values within columns followed by different lowercase letters are significantly different at *P* < 0.05 according to least-significant-difference test.

## Abbreviations

CF: Conventional flooding
Chl: Chlorophyll
*C*_*i*_: Intercellular CO_2_ concentration
DI: Drip irrigation with plastic mulching
DMPA: Dry matter postanthesis
DMPrA: Dry matter preanthesis
*E*: Transpiration rate
FIM: Furrow irrigation with plastic mulching
FIN: Furrow irrigation with no mulching
*F*_*m*_: Maximal fluorescence yield of dark-adapted state
*F*_*m*_′: Maximal fluorescence yield of light-adapted state
*F*_*o*_: Minimum fluorescence yield of dark-adapted state
*F*_*o*_′: Minimal fluorescence yield of light-adapted state
*F*_*s*_: Steady-state fluorescence yield
*F*_*v*_/*F*_*m*_: Maximal quantum yield of PSII photochemistry
*F*_*v*_′/*F*_*m*_′: PSII maximum efficiency
FW: Fresh weight
*g*_*s*_: Stomatal conductance
LAI_max⁡_: Leaf area index at flowering stage
NLA: Nitrogen content per leaf area
NPQ: Non-photochemical quenching
*P*_*N*_: Net photosynthetic rate
*q*_*P*_: Photochemical quenching coefficient
Φ_PSII_: Effective quantum yield of PSII photochemistry
SE: Standard error
TNC: Total nitrogen content
TTC: Triphenyltetrazolium chloride.
